# Investigating the Interaction of an Anticancer Nucleolipidic Ru(III) Complex with Human Serum Proteins: A Spectroscopic Study

**DOI:** 10.3390/molecules28062800

**Published:** 2023-03-20

**Authors:** Claudia Riccardi, Antonella Campanella, Daniela Montesarchio, Pompea Del Vecchio, Rosario Oliva, Luigi Paduano

**Affiliations:** 1 Department of Chemical Sciences, University Federico II of Napoli, Via Cintia 21, 80126 Napoli, Italy; 2 CINMPIS—Consorzio Interuniversitario Nazionale di Ricerca in Metodologie e Processi Innovativi di Sintesi, Via E. Orabona 4, 70125 Bari, Italy; 3 CSGI—Consorzio Interuniversitario per Lo Sviluppo dei Sistemi a Grande Interfase, Via della Lastruccia 3, 50019 Florence, Italy

**Keywords:** ruthenium(III) complexes, anticancer drugs, liposomes, serum proteins, interactions

## Abstract

Ruthenium(III) complexes are very promising candidates as metal-based anticancer drugs, and several studies have supported the likely role of human serum proteins in the transport and selective delivery of Ru(III)-based compounds to tumor cells. Herein, the anticancer nanosystem composed of an amphiphilic nucleolipid incorporating a Ru(III) complex, which we named DoHuRu, embedded into the biocompatible cationic lipid DOTAP, was investigated as to its interaction with two human serum proteins thought to be involved in the mechanism of action of Ru(III)-based anticancer drugs, i.e., human serum albumin (HSA) and human transferrin (hTf). This nanosystem was studied in comparison with the simple Ru(III) complex named AziRu, a low molecular weight metal complex previously designed as an analogue of NAMI-A, decorated with the same ruthenium ligands as DoHuRu but devoid of the nucleolipid scaffold and not inserted in liposomal formulations. For this study, different spectroscopic techniques, i.e., Fluorescence Spectroscopy and Circular Dichroism (CD), were exploited, showing that DoHuRu/DOTAP liposomes can interact with both serum proteins without affecting their secondary structures.

## 1. Introduction

Interest in Ru-based anticancer agents has rapidly increased since the discovery of their promising anticancer activity coupled with a significantly lower toxicity compared to known Pt-containing drugs [1,2,3,4,5,6,7].

Three Ru(III)-containing complexes have undertaken advanced clinical trials as anticancer drugs, i.e., NAMI-A [8,9], KP1019 [10,11,12,13] and its sodium salt analogue known as NKP-1339 [14,15,16,17]. Despite their structural similarity (Supplementary Materials, Figure S1), these Ru(III) complexes significantly differ in their bioactivity [18], but share the ability to interact with plasma proteins, particularly serum albumin [19,20,21,22,23,24,25], by far the most abundant protein in the plasma, and human transferrin (hTf) [26,27,28,29,30]. In this context, particularly promising were also Ru(III) complexes bearing tetradentate N_2_O_2_ bis(aminophenolate) ligands, i.e., Salan-type ligands, which exhibited significant cytotoxicity against different cancer cell lines and a strong binding to serum albumin, as determined by steady-state and time-resolved fluorescence spectroscopy [31]^.^ In addition, they were recently proved to strongly interact with lipid bilayers, mimicking mammalian plasma or cancer cell line membrane, leading to a significant increase in the permeability of the lipid vesicles without significantly affecting their structure [32].

**Scheme 1:**
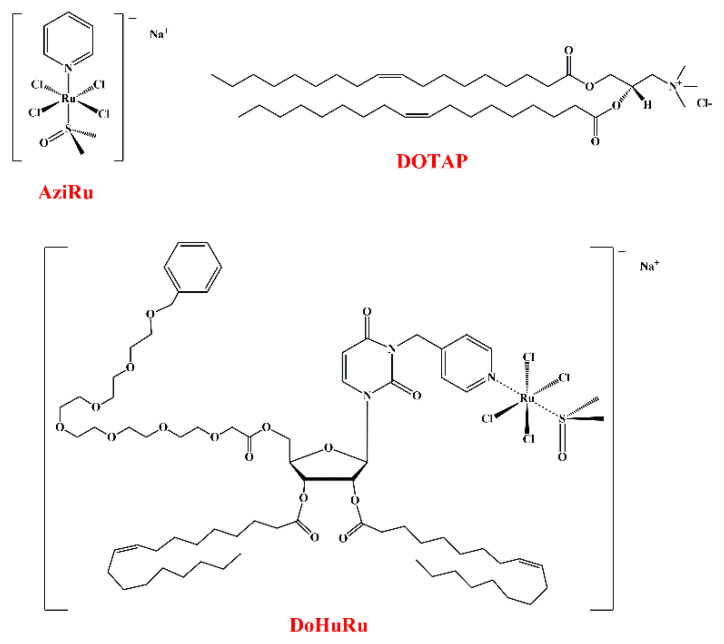
Chemical structures of the Ru(III) complex AziRu, the cationic lipid DOTAP and nucleolipid-based Ru(III) complex DoHuRu, as indicated.

Intrigued by the promising properties of these Ru(III)-based anticancer agents, a NAMI-A-like Ru(III) complex—first described by Attia et al. in 1993[33] and then revisited by Walsby and colleagues [34] and by some of us [35,36]—was considered a valuable starting compound for further optimization. From a structural point of view, this low molecular weight complex, which we named AziRu (Scheme 1), has a pyridine ligand replacing the imidazole moiety of NAMI-A and sodium in place of imidazolium as the counterion. Like NAMI-A, AziRu proved to be poorly cytotoxic on various tumorigenic cells, showing lower IC_50_ values, however, on human breast MCF-7 and cervical HeLa cancer cell lines than its parent compound (Supplementary Materials, Table S1) [35,37].

Analogously to NAMI-A, AziRu undergoes a hydrolysis process in aqueous solutions [38] through a ligand exchange mechanism, mainly involving the replacement of the more labile chloride ligands with water molecules or hydroxide ions, providing more reactive aquo complexes, eventually leading to poly-oxo species formation [39,40,41,42,43].

A detailed insight into the interaction of AziRu with proteins in the solid state could be obtained by analyzing crystals of its complexes with bovine pancreatic ribonuclease A (RNase A) [44] and hen egg white lysozyme (HEWL) [45,46]. In both cases, upon binding, the metal lost all its original ligands, as a consequence of the hydrolysis process, but the protein structure was not significantly altered by the metal complexation [44,45].

Although it is generally accepted that the hydrolysis process activates Ru(III) complexes into more reactive species effectively able to react with potential biomolecular targets, such as proteins or nucleic acids, the premature formation of aquated or poly-oxo species in the extracellular medium can deactivate, or activate too early, most of the administered drug. Thus, the hydrolysis of Ru(III) complexes must be retarded, not to be reached until at least after they are effectively internalized into cells [47].

To this end, AziRu was decorated with nucleolipid [47,48] or aminoacyl lipid scaffolds [49], and then co-aggregated with vesicle-forming lipids as the zwitterionic POPC (1-palmitoyl-2-oleoyl-sn-glycero-3-phosphocholine) or the cationic DOTAP (1,2-dioleoyl-3-trimethylammoniumpropane) (Scheme 1), providing a mini-library of various Ru(III)-loaded nanovectors [47]. The use of highly functionalized scaffolds combined with their co-formulation with biocompatible lipids proved to efficiently increase the stability of AziRu in physiological conditions, by reducing its hydrolysis rate, and also to enhance its cellular internalization [35,36,37,50,51,52,53,54]. As a major achievement, the proposed design allowed converting a weakly cytotoxic compound, i.e., AziRu, into potent antiproliferative agents, particularly active on breast cancer cells, without showing significant toxicity on healthy cell lines [35,36,37,47,50,51,52,53,54]. These formulations proved to be effective and selective also in vivo in breast cancer xenografts mouse models [55,56].

**Scheme 2:**
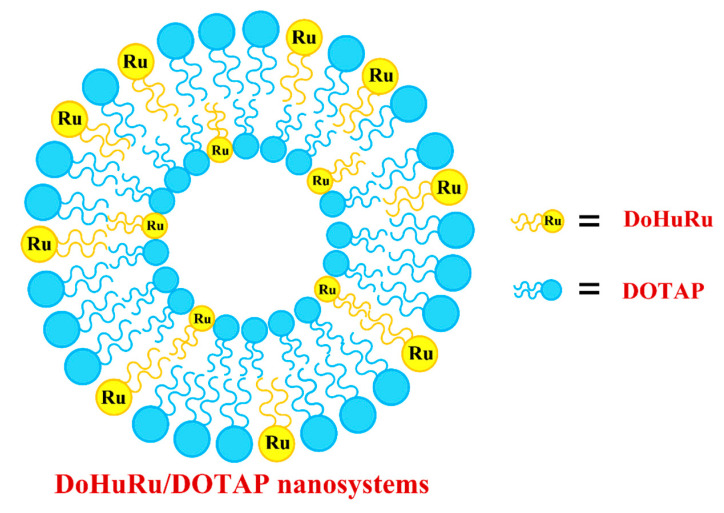
Schematic representation of liposomal DOTAP-based nanoaggregates containing the nucleolipid-based Ru(III) complex DoHuRu.

Among the various developed systems, the nucleolipid-based Ru(III) complex called DoHuRu (Scheme 1) [35,51,52], embedded into the biocompatible lipid DOTAP (a schematic representation of the obtained liposome-based nanosystem is reported in Scheme 2) proved to have intriguing biological activity on different cancer cell lines with respect to naked AziRu (Supplementary Materials, Table S1), and was chosen here as a representative compound in the previously prepared compounds library to analyze in more detail their binding properties with serum proteins.

Since anticancer Ru(III)-based compounds are usually administered intravenously, extensive in vivo binding to plasma proteins can have significant consequences on the biodistribution and bioavailability of these complexes [57]. These proteins can in fact behave as suitable carriers for ruthenium inside cells through permeation and/or endocytosis mechanisms, providing selective tumor targeting [58,59,60,61,62].

In detail, human serum albumin (HSA) is the major soluble protein constituent of the circulatory system, identified as a well-established transporter of both endogenous and exogenous species in the bloodstream, including drugs [63,64]. In addition, HSA is known to accumulate in tumors as a consequence of enhanced permeability and retention effect [60,61,62].

In turn, to promote their rapid growth, some malignant cells require high levels of iron(III) and for this reason upregulate the expression of transferrin receptors on their surface. In this context, human transferrin (hTf) could have a role in vivo as a natural carrier for metal complexes, and hTf receptors can be exploited to provide a privileged route for delivering drugs to tumor cells [59,65].

Considering that both albumin and transferrin are involved in the mechanism of action proposed for NAMI-A, in this work we studied the interaction of the effective anticancer DoHuRu/DOTAP formulations with HSA and hTf, using the naked AziRu complex and the pure lipid DOTAP as controls.

Aiming at obtaining precious information on the mechanism of action of the proposed liposome-containing nucleolipid-based Ru(III) complexes, fluorescence and Circular Dichroism (CD) spectroscopies data were exploited here. In detail, fluorescence spectroscopy was used to obtain information on the interaction between the proteins and AziRu or the Ru(III)-containing vesicles. In contrast, CD measurements were exploited to verify the effect on the secondary structures of the target proteins produced by binding with AziRu or DoHuRu/DOTAP liposomes.

## 2. Results and Discussion

### 2.1. Preparation and Characterization of Ru(III) Complexes and Liposomes

As a first step, the Ru(III) complexes AziRu [34,38] and DoHuRu [35,51,52] were synthesized following previously described procedures. Then, DoHuRu/DOTAP liposomes were prepared through the thin film protocol by dissolving known weighed amounts of both components and mixing them at the desired Ru complex: lipid 30:70 molar ratio. In parallel, also bare DOTAP liposomes were prepared as control. After preparation, as described in Material and Methods and in Mangiapia et al. [51], these liposomes were analyzed by Dynamic Light Scattering (DLS) measurements. In Supplementary Materials, Figure S2, the hydrodynamic radius distribution functions for the two preparations are shown. The hydrodynamic radius of the DOTAP and DoHuRu/DOTAP nanosystems proved to be in the 70–100 nm range, which is the typical range of unilamellar vesicles, in accordance with previous investigations [51]. DLS measurements fully confirmed a monodisperse liposome dispersion for both bare DOTAP and DoHuRu/DOTAP formulations.

### 2.2. Study of the Interaction of AziRu and DoHuRu/DOTAP Liposomes with Serum Proteins

Fluorescence quenching is a widely used technique to gain information on the interaction of serum proteins with drugs or small ligands [66,67]. The fluorescence emission of proteins comes from the three aromatic residues tryptophan, tyrosine, and phenylalanine. Phenylalanine has a very low quantum yield, and the fluorescence of tyrosine is almost totally quenched if it is ionized or close to an amino group, a carboxyl group, or a tryptophan. Hence, the fluorescence of proteins (and thus also of HSA and hTf) is almost exclusively due to the tryptophan emission alone. Particularly, the emission spectrum of HSA mainly derives from a single tryptophan residue located at the 214 position in the subdomain IIA, while that of hTf is essentially due to 8 Trp residues. In addition to the common sources of quenching, in the case of hTf, the two iron ions strongly affect the hTf emission spectrum [68]. Changes in the intrinsic fluorescence intensity of HSA and hTf proteins—due to spatial reorganization of the tryptophan residues—are evidenced when small molecules are bound to the proteins [69].

**Figure 1:**
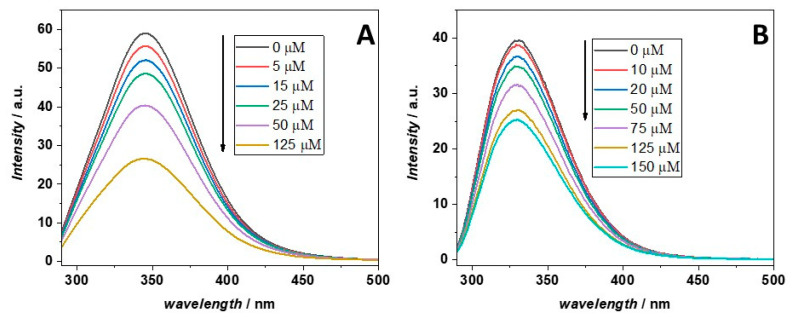
Fluorescence emission spectra at 20 °C of (**A**) HSA and (**B**) hTf in the absence and presence of increasing concentrations of AziRu, as reported in the legend. The spectra of the proteins in the absence of the ligand are reported in black. The concentrations of HSA and hTf were kept constant at 5 μM. The excitation wavelength was set at 280 nm. The fluorescence emission of HSA and hTf are centered at 345 and 330 nm, respectively. The arrows indicate the direction of the effects due to addition of increasing concentrations of AziRu.

The nude AziRu complex has been used as a reference in this study to probe the effects of the liposomal carrier in interactions with human serum proteins. The fluorescence spectra at 20 °C of HSA and hTf in the absence and presence of AziRu are reported in Figure 1A,B, respectively.

Upon excitation at 280 nm, HSA and hTf showed strong fluorescence emission bands centered at 345 and 330 nm, respectively. On the other hand, AziRu has no intrinsic fluorescence upon excitation at the same wavelength. It is important to note that AziRu absorbs at the used excitation wavelength and, to a much lesser extent, at the wavelength of emission of the proteins (see Supplementary Materials, Figure S3 for the UV/Vis spectrum of AziRu in PBS buffer). Thus, an inner filter effect could be active and should be corrected. However, since at the used AziRu concentrations, the absorbance of the ligand is quite low, the lack of correction can lead to a slight overestimation of the binding constants. The addition of increasing amounts of AziRu (in the 5–150 μM range) to each protein, kept at a fixed concentration of 5 μM, produced a marked fluorescence quenching, that represents clear evidence of interaction. Similar outcomes were reported for several Ru complexes [70,71,72], and also for the binding of NAMI-A to hTf [28,29] where, in all cases, a decrease of fluorescence intensity was observed upon binding. It is important to note that the observed decrease of intensity suggests that the Trp residues are localized not far from the binding pocket of AziRu, otherwise no changes in the intensity could be observed. Finally, the binding of AziRu to both proteins did not cause any change in the emission maximum and shape of the peaks, indicating that upon binding, the microenvironment surrounding the Trp residues was not affected by the small ligand.

**Figure 2:**
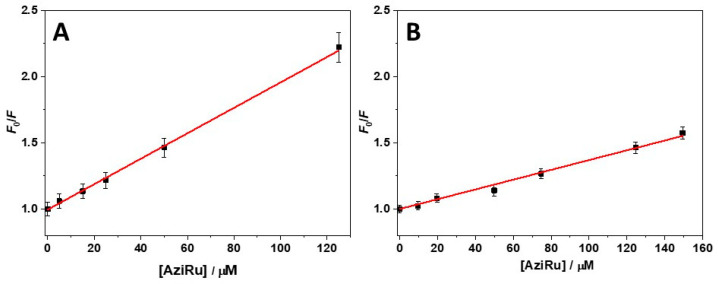
Stern–Volmer plots for the binding of AziRu with (**A**) HSA and (**B**) hTf at 20 °C. The red lines represent the best fit of experimental data according to equation 1 reported in the text.

To evaluate the strength of the complex formed between AziRu and each serum protein, fluorescence quenching data were analyzed by the Stern–Volmer Equation (1):

(1) $$\frac{F_{0}}{F}=1+K_{SV}\left[Q\right]=1+K_{S}\left[Q\right]$$

where *F*_0_ and *F* are the steady-state fluorescence intensities of the proteins in the absence and presence of the quencher AziRu, respectively. *K_SV_* is the Stern–Volmer quenching constant and [*Q*] is the concentration of the quencher, i.e., AziRu in the present case. As will be shown later, the quenching mechanism was found to be static. Thus, the Stern–Volmer constant is, in this case, a quenching constant due to a static process (i.e., complex formation) and, thus, can be indicated also as *K_S_* (where *S* is for static). In Figure 2, the Stern–Volmer plots obtained at the temperature of 20 °C for the titration of a solution of HSA and hTf with a solution of AziRu are reported.

As evidenced by the data reported in Figure 2, a linear plot was obtained in both cases, allowing the data fitting by using equation 1. The obtained values of *K_SV_* for the interaction of AziRu with HSA and hTf are (9.7 ± 0.5) × 10^3^ M^−1^ and (3.7 ± 0.7) × 10^3^ M^−1^, respectively (Table 1). These data revealed a higher ability of AziRu to quench the fluorescence of HSA than that of hTf, suggesting a higher affinity of AziRu for HSA than hTf. However, quenching due to the interaction of AziRu with proteins can be dynamic (deactivation of the excited state through collisional events) or static (complex formation in the ground state) in nature [73]. Static and dynamic quenching can be distinguished on the basis of their dependence from temperature. Higher temperatures result in faster diffusion and hence a larger amount of collisional quenching. Thus, if dynamic quenching is the only active mode, an increase in temperature should lead to an increase of the value of *K_SV_*. In contrast, when the quenching is static, an increase in temperature should not favor the complex formation, and a decrease of *K_SV_* is normally observed [73]. In Supplementary Materials, Figure S4, the Stern–Volmer plots recorded at 10 and 20 °C are shown. In Table 1 the corresponding values of *K_SV_* are collected.

As reported in Table 1, the value of *K_SV_* decreased on increasing the temperature for both proteins, i.e., *K_SV_* (20 °C) < *K_SV_* (10 °C), thus indicating that the quenching mechanism is essentially static. Finally, in supporting the static nature of the observed decrease in fluorescence, it is to be considered that the *K_SV_* values for dynamic quenching are usually ~10^1^ M^−1^. In contrast, for static quenching, the constants are higher at least by one order of magnitude. Thus, one can conclude that the observed quenching is indeed due to the formation of a complex between the ligand and the proteins. Consequently, the value of *K_SV_* is equivalent to the binding constant (*K_b_*), defined as the ratio between the complex concentration and the product of the concentrations of the free ligand and free protein. Collectively, the reported data showed that AziRu had a higher affinity for HSA compared to hTf. However, it is important that both obtained *K_SV_* values are quite small. This finding agrees with the hypothesized role of these proteins in serum, allowing the transport of small compounds, but also their release.

The static nature of the quenching mechanism, as demonstrated above, allows us treating the Stern–Volmer constants as binding constants for the complex formation. However, in the Stern–Volmer analysis, a 1:1 complex is assumed to be formed. The linearity of the Stern–Volmer plots reported in Figure 2 is strong evidence that the stoichiometry is effectively 1:1, otherwise deviations from linearity should be observed. However, in order to determine the binding stoichiometry (*n*) and further support the 1:1 complex formation, the Stern–Volmer equation can be rearranged in the following form (Equation (2)) [74]:

(2) $$\log\left(\frac{F_{0}-F}{F}\right)=\log K_{b}+n\log\left[Q\right]$$

where *F*_0_ and *F* are the fluorescence intensities in the absence and presence of the ligand, respectively, [*Q*] is the ligand concentration, *K_b_* is the apparent binding constant, and *n* is the stoichiometry defined as the number of ligand molecules interacting with one molecule of protein. The calculated *K_b_* and *n* at 20 °C are reported in Table 2. In turn, the corresponding plots of log(*F*_0_ −*F*/*F*) vs. log[Q] are shown in Supplementary Materials, Figure S5.

The obtained values showed that both proteins formed a 1:1 complex, with obtained values of *K_b_* in excellent agreement with the values of *K_SV_* reported above, completely validating the Stern–Volmer analysis reported above [75].

Next, in order to probe specific interactions between the studied proteins and the Ru-containing vesicles, the same fluorescence analysis carried out on AziRu was performed with the DoHuRu/DOTAP liposomes. However, to take into account possible interactions between the proteins and pure DOTAP vesicles, both protein solutions were titrated first with a pure DOTAP vesicles suspension, and, after each addition, the corresponding fluorescence spectrum was acquired. The spectra obtained for HSA/DOTAP and hTf/DOTAP systems are shown in Supplementary Materials, Figure S6A and S6B, respectively.

For both proteins, a small but significant decrease of their fluorescence intensity was observed upon addition of DOTAP vesicles. In particular, at the highest DOTAP concentration tested, the fluorescence intensity drop in the case of HSA was around 16% and for hTf around 5%. In addition, no wavelength shift of the fluorescence maximum (346 nm for HSA and 332 nm for hTf) was observed. The reported data indicated that both proteins could interact with DOTAP vesicles. However, due to the low change in the fluorescence intensity, the interaction appeared overall very weak, particularly in the case of hTf.

**Figure 3:**
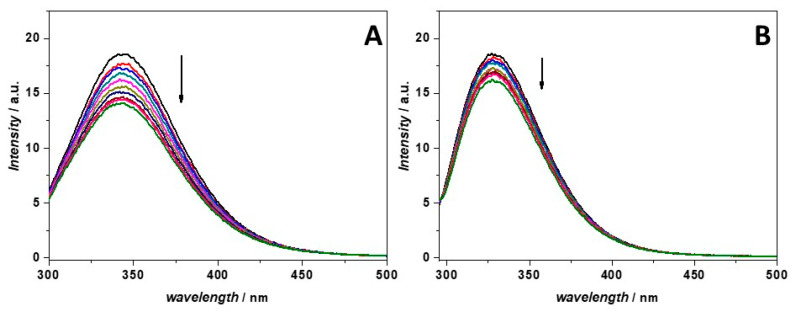
Fluorescence emission spectra of (**A**) HSA and (**B**) hTf in the absence (black spectra) and in the presence of increasing DoHuRu/DOTAP vesicles concentrations (colored spectra). The arrows indicate the direction of the effects due to increasing concentrations of DoHuRu/DOTAP. The excitation wavelength was set at 280 nm. All the experiments were performed in a 1-cm path length quartz cuvette at the temperature of 20 °C.

The same titration experiments were repeated by titrating the HSA and hTf solutions with the DoHuRu/DOTAP 30:70 vesicles suspension (Figure 3A and Figure 3B, respectively).

For the HSA titration (Figure 3A), a marked decrease of the fluorescence intensity upon addition of the Ru-containing vesicles was observed, reaching a 24% fluorescence intensity drop at the highest vesicle concentration explored. In contrast, the decrease in fluorescence intensity at 332 nm observed for hTf after titration with the Ru-containing vesicles was less pronounced (around 12%). The observed decrease could be due to the complex formation between the proteins and the vesicles, which affects the fluorescence emission of the tryptophan residues. In addition, in both cases, no shift of the emission maximum was observed, indicating that the microenvironment surrounding the Trp residues was not affected by the interaction process, thus suggesting that the interaction probably occurred without any conformational change of the proteins.

In order to quantitatively describe the complex formation with the Ru-containing vesicles, these data were treated by the Stern–Volmer analysis as carried out with AziRu. The Stern–Volmer plots obtained from the titrations experiments on the serum proteins with DOTAP and DoHuRu/DOTAP are reported in Supplementary Materials, Figure S7, while the values of the corresponding Stern–Volmer constants are reported in Table 3.

Inspection of the data reported in Table 3 revealed that HSA is able to interact with DOTAP vesicles alone. This agrees with the known ability of serum proteins like BSA and HSA to interact with a wide variety of ligands, and primarily fatty acids [76]. When DOTAP was coformulated with the nucleolipid-based Ru(III) complex DoHuRu, an increase of the *K_SV_* value was observed (from 2.9 × 10^3^ M^−1^ to 5.5 × 10^3^ M^−1^), clearly demonstrating that the interaction of liposomes with proteins is enhanced when the nucleolipidic Ru complex is incorporated in the vesicles. It is interesting to note that the presence of a lipid formulation does not seem to dramatically limit the ability of HSA to interact with the Ru complex, since the *K_SV_* value for DoHuRu/DOTAP vesicles is only ca. two-fold lower with respect to that found with AziRu.

As far as hTf is concerned, this protein proved to be able to interact with pure DOTAP vesicles, but with a lower affinity with respect to HSA (*K_SV_* of 0.98 × 10^3^ M^−1^ vs. 2.9 × 10^3^ M^−1^). Interestingly, the vesicles containing the Ru complex showed enhanced ability to interact with hTf compared to pure DOTAP liposomes, also highlighting in this case the presence of specific interactions with the Ru complex. Indeed, the value of *K_SV_* for the complex between hTf and DoHuRu/DOTAP liposomes (2.3 × 10^3^ M^−1^) was comparable, within the experimental error, to the one obtained for AziRu (3.7 × 10^3^ M^−1^).

Finally, the hydrodynamic radius distribution of DOTAP and DoHuRu/DOTAP vesicles in the presence of the two serum proteins was also verified by means of dynamic light scattering experiments. In Supplementaty Materials, Figures S8 and S9 the hydrodynamic radius distribution functions for vesicles in the absence and presence of HSA and hTf are reported, respectively. HSA affects the size distribution of DOTAP and DoHuRu/DOTAP vesicles in a concentration dependent manner, leading to a broader size distribution centered at higher R_H_ values compared to those in the absence of the protein. Interestingly, this effect is evident for DoHuRu/DOTAP vesicles at a lower protein concentration with respect to the bare DOTAP, which could be attributable to the higher affinity of HSA for the nucleolipid Ru complex. In contrast, the size distribution of DOTAP vesicles is not affected at all by the presence of hTf. This is expected for a low affinity binding event. Surprisingly, hTf showed a marked effect on DoHuRu/DOTAP vesicles. As in the case of HSA, at the highest protein concentration used, the size distribution is centered at higher R_H_ values compared to those in the absence of protein, leading to bigger vesicles. However, it is remarkable that the integrity of liposomes is always preserved upon proteins binding.

### 2.3. Conformational Behavior of Serum Proteins in the Presence of the Ru-Containing Complexes

**Figure 4:**
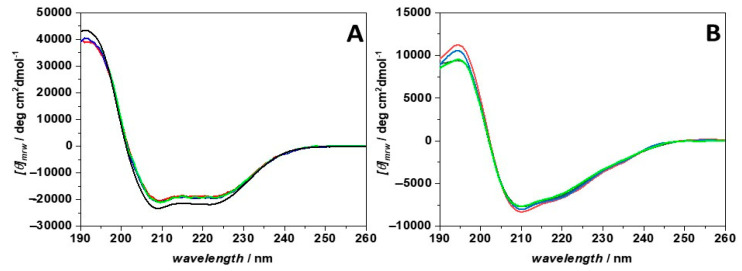
Far UV-CD spectra of (**A**) HSA and (**B**) hTf recorded in the absence (black spectra) and in the presence of increasing concentrations of AziRu. The concentrations of both proteins were 1.5 μM. The AziRu concentrations were: 1.5 μM (red spectra), 7.5 μM (blue spectra) and 75 μM (green spectra). The spectra were recorded at 20 °C by using a 0.1 cm path length quartz cuvette.

Changes in the protein secondary structure induced by ligand binding and/or interaction with vesicles are typically studied by recording CD spectra in the far-UV region [77]. The CD spectra of free HSA and hTf in the absence and presence of increasing concentrations of AziRu are shown in Figure 4A and Figure 4B, respectively.

The CD spectrum of HSA showed two negative bands at 208 and 220 nm, and a positive band around 195 nm, indicative of the presence of an α-helix structure [77], in agreement with previously reported CD data and the deposited protein crystal structure [76,78]. Conversely, the CD spectrum of hTf showed a distinct minimum around 210 nm, a weak negative band around 220 nm, and a positive band at 195 nm. These spectral features suggested that also this protein adopts an α-helical conformation. However, the intensities of the two minima are not the same, indicating that not all the protein is folded in a helix conformation. Indeed, an inspection of the hTf crystal structure revealed the presence of a consistent portion of the protein folded in a twisted β-sheet conformation, as well as turns and disordered regions [79], thus explaining the obtained CD spectrum.

For HSA, in the presence of AziRu (at a ligand-to-protein ratio of 1:1, 5:1, and 50:1), only a modest decrease in the intensity of these negative bands occurred, suggesting that the interaction with this small ligand does not sensibly affect the overall secondary structure of the protein. Similar results were obtained for hTf. Thus, even if AziRu can bind both proteins, the integrity of their secondary structures is preserved upon complex formation.

**Figure 5:**
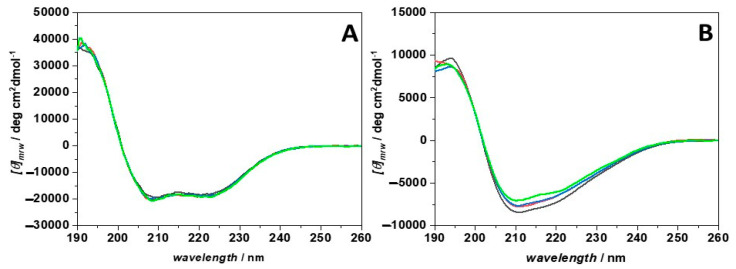
Far UV-CD spectra of (**A**) HSA and (**B**) hTf recorded in the absence (black spectra) and in the presence of increasing concentrations of DoHuRu/DOTAP. The concentrations of both proteins were 1.5 μM. For HSA, the vesicles concentrations were 45 μM (red spectrum), 75 μM (blue spectrum), and 150 μM (green spectrum). For hTf, vesicles concentrations were 1.5 μM (red spectrum), 15 μM (blue spectrum) and 75 μM (green spectrum). The spectra were recorded at 20 °C by using a 0.1 cm path length quartz cuvette.

The same experiments were also performed in the presence of the DoHuRu/DOTAP liposomes, to verify possible conformational changes of the proteins imposed by the complexation with Ru(III) containing vesicles. In Figure 5, the CD spectra of HSA (panel A) and hTf (panel B) in the absence and presence of increasing concentrations of DoHuRu/DOTAP are shown.

Interestingly, the addition of the vesicles to HSA did not affect at all the secondary structure of the protein, even at the very high vesicles/protein concentration ratio of 100:1. This result agrees with previous studies showing that the secondary structure of HSA is only slightly affected by interaction with vesicles [80,81]. In sharp contrast, the CD spectra recorded for hTf suggested that some, even if small, conformational changes are produced in the protein upon interaction with DoHuRu/DOTAP vesicles. Indeed, the intensities of the two negative bands had already decreased at the lowest vesicle concentrations investigated (1.5 and 15 μM, i.e., at a 1:1 and 10:1 vesicles/protein concentration ratio). Upon increasing the vesicles concentration at 75 μM, the changes were more evident. Conversely, the intensity of the positive band at 195 nm was not affected by the interaction process. These findings suggested that the helical content of the protein decreased upon interaction, but to a low extent. Conversely, the CD spectrum of hTf (Supplementary Materials, Figure S10) was not affected at all by the presence of DOTAP vesicles. This result highlights the key role played by the nucleolipid-based Ru complex in driving the interaction process, inducing small conformational changes in the protein structure. In conclusion, the overall secondary structure of hTf is only marginally affected by the presence of DoHuRu/DOTAP vesicles.

## 3. Experimental Section

### 3.1. Materials and Methods

The following were all purchased from Sigma Aldrich and used as received: 1,2-dioleoyl-3-trimethylammonium-propane (chloride salt) (DOTAP, MW = 698.55 g/mol, >99%); albumin from human serum (≥99%); transferrin human (≥98%); and chloroform (for HPLC, ≥99.9%). All the experiments were carried out in a pseudo-physiological phosphate buffer solution (NaCl = 137 mM, KCl = 2.7 mM, Na_2_HPO_4_ = 10 mM, KH_2_PO_4_ = 2 mM, pH = 7.4).

### 3.2. Synthesis of AziRu and DoHuRu Ru(III) Complexes

AziRu was prepared by reacting pyridine with Na^+^ [*trans*-RuCl_4_(DMSO)_2_]^−^ in anhydrous acetonitrile, following a previously described protocol [34,38]. The nucleolipidic ruthenium complex, named DoHuRu, was prepared by reacting in stoichiometric amounts the starting nucleolipid, named DoHu [48], with the Ru complex Na^+^ [*trans*-RuCl_4_(DMSO)_2_]^−^ following previously reported procedures [35,51,52].

The desired salt was obtained in almost quantitative yields and in pure form, as confirmed by TLC, NMR, and ESI-MS analysis, whose results were in all cases in accordance with literature data.

FTIR-ATR of AziRu: significant vibrational frequencies (cm^−1^): 3018. 2 (w) [*ν* (*CH*)], 1667.1 (s) [*ν* (*C=N*)], 1317.2 (w) [*δ* (*CH*)]; 1080.3 (s) [*ν* (*S=O*) (*DMSO*)]; 1025.8 (s) [*ρ* (*CH*)]; 764.0 (s) [*ν* (*C-S*)].

### 3.3. Preparation of DOTAP and DoHuRu/DOTAP Vesicles

For the preparation of the vesicles composed of 1,2-dioleoyl-3-trimethylammonium-propane (chloride salt) (DOTAP), 0.346 mL (0.00101 mmol) of a DOTAP standard solution in chloroform (2.92 mM) was transferred in a round-bottom glass vial. A thin film was obtained through evaporation of the solvent under a gentle flow of dry N_2_ for at least 24 h. The film was hydrated with the phosphate buffer and dispersed in solution through sonication at 59 kHz at 40 °C for at least 40 min. The vesicles suspension was then extruded through polycarbonate membranes of 100 nm pore size, at least 11 times. For the preparation of vesicles composed of DoHuRu and DOTAP, 2.47 mL (0.000721 mmol) of a DoHuRu (MW = 1620 g/mol) standard solution (0.292 mM), and 0.592 mL (0.00179 mmol) of a DOTAP standard solution (3.02 mM) were introduced in a round bottom glass vial. Then, the following steps were identical to the ones used for the preparation of bare DOTAP liposomes.

### 3.4. Preparation of the Protein Solutions

The solutions of HSA (M.W. 66 kDa g mol^−1^) and hTF (M.W. 80 kDa) were prepared by dissolving the lyophilized powder directly in PBS buffer, pH 7.4. Their concentrations were determined spectrophotometrically by evaluating their absorbance at 280 nm, using the following extinction coefficient at 280 nm: ε(280) = 35700 M^−1^ cm^−1^ and ε(280) = 85240 M^−1^ cm^−1^ for HSA and hTf, respectively.

### 3.5. Dynamic Light Scattering Experiments

Dynamic light scattering (DLS) analyses were performed with a setup composed of Photocor compact goniometer, a SMD 6000 Laser Quantum 50 mW light source operating at 5325 Å, and a PMT and correlator obtained from Correlator.com. All the measurements were performed at (25.00 + 0.05) °C by using a thermostat bath. The concentration of the used lipids was 0.5 mM in all the performed experiments. The concentrations of HSA were 0.12 µM and 0.45 µM. In turn, the concentrations of hTf were 0.15 µM and 0.45 µM. DLS measurements were performed 24 h after each addition of protein solution into the vesicles suspension.

### 3.6. Fluorescence Spectroscopy Experiments

The interaction between serum proteins HSA and hTf with AziRu, DOTAP and DoHuRu/DOTAP vesicles was studied by following the intrinsic fluorescence changes of proteins induced by the addition of each compound. The titrations were performed by recording spectra of a protein solution at fixed concentration (5 μM) containing increasing Ru(III) complex or DOTAP amounts ranging from 0 to ~10^−4^ M. AziRu stock solutions were prepared by weight. All the samples were prepared in a saline sodium phosphate buffer (PBS) solution at pH 7.4.

Steady state fluorescence measurements were performed by means of a FluoroMax-4 fluorescence spectrophotometer (Horiba, Edison, NJ, USA), using a quartz micro cuvette (1 cm in excitation and 0.2 cm in emission) of 400 μL volume. The excitation wavelength was 280 nm, and the emission spectra were recorded in the 290–500 nm range. The excitation and emission slits were 3 nm, and the temperature was set to 20 °C and 10 °C. All protein fluorescence spectra were corrected by subtraction of the buffer alone.

### 3.7. Circular Dichroism Experiments

Circular dichroism (CD) spectra were acquired in the far UV-region (190–250 nm) using a Jasco J-715 spectropolarimeter (from Jasco Corporation, Tokyo, Japan) under constant nitrogen flow. The temperature was controlled by a Peltier type temperature control system (Model PTC-348WI). The spectra were recorded in a 0.1 cm quartz cuvette, with 4 s response time, 2 nm bandwidth, and 20 nm/min scan rate. Protein concentrations between 0.05–0.1 mg/mL were typically used. The spectra were measured in the absence and presence of increasing concentrations of AziRu or DoHuRu/DOTAP vesicles in a 20 mM sodium phosphate buffer, pH 7.4. Spectra were recorded at a fixed temperature of 20 °C, averaged, and corrected for the buffer baseline.

## 4. Conclusions

With the aim of better understanding the mechanism of action of Ru(III)-based anticancer drugs, we herein investigated via spectroscopic measurements the interaction occurring between the nucleolipidic Ru complex DoHuRu embedded in DOTAP liposomes and two serum proteins, i.e., HSA and hTf, using the NAMI-A-like low molecular weight complex AziRu as a reference.

Fluorescence analysis gave solid evidence of the binding of the DoHuRu/DOTAP liposomes, and of AziRu as well, with both proteins. Indeed, in all cases the addition of the Ru(III)-containing compounds to the target proteins caused a quenching of the protein intrinsic fluorescence, which increased on increasing the amount of ligand, indicative of the formation of stable ligand-protein complexes. Notably, the quantitative analysis of the fluorescence data revealed binding constants in the 10^3^–10^4^ M^−1^ range, indicative of a moderate affinity. This is fully consistent with the postulated behaviour of these proteins, able to sequester anticancer metal-based drugs in the bloodstream and then release them in the proximity of tumour tissues. These fluorescence analysis data were further corroborated by CD spectroscopy.

Circular dichroism spectra of the studied proteins were essentially similar in shape if registered in the absence or presence of each complex, suggesting that the binding did not alter their secondary structures.

Taken together, our results highlight the fact that DoHuRu/DOTAP liposomes can also interact extensively with serum proteins, similarly to NAMI-A-like complex AziRu and in line with other anticancer Ru(III) complexes described in the literature. This binding can have important consequences for the pharmacokinetics of these metal complexes. Indeed, the formation of Ru(III) complex/serum protein adducts is of potential pharmaceutical interest, considering the putative in vivo role of these proteins as carriers allowing the selective delivery of metal complexes to cancer cells and tissues.

## Figures and Tables

**Table 1:** Stern–Volmer constants for the complex formation between AziRu and serum proteins obtained at 10 °C and 20 °C.

System	T/°C	*K_SV_*/M^−1^
HSA-AziRu	10	(1.1 ± 0.5) × 10^4^
HSA-AziRu	20	(9.7 ± 0.5) × 10^3^
hTf-AziRu	10	(5.1 ± 1.0) × 10^3^
hTf-AziRu	20	(3.7 ± 0.7) × 10^3^

**Table 2:** Values of the apparent binding constant (*K_b_*) and stoichiometry (*n*) for the complex formation between AziRu and each serum protein obtained at 20 °C.

System	*K_b_*/M^−1^	*N*
HSA-AziRu	(1.2 ± 0.3) × 10^4^	0.94 ± 0.20
hTf-AziRu	(2.3 ± 0.5) × 10^3^	1.1 ± 0.2

**Table 3:** Stern–Volmer constants for the complex formation between DOTAP and DoHuRu/DOTAP vesicles with serum proteins (HSA and hTf) obtained at 20 °C.

Scheme 1	T/°C	*K_SV_*/M^−1^
HSA-DOTAP	20	(2.9 ± 0.7) × 10^3^
HSA-DoHuRu/DOTAP	20	(5.5 ± 0.5) × 10^3^
hTf-DOTAP	20	(0.98 ± 0.10) × 10^3^
hTf-DoHuRu/DOTAP	20	(2.3 ± 0.6) × 10^3^

## Data Availability

All the data are available on request from the corresponding author.

## Supplementary Materials

The following are available online at https://www.mdpi.com/article/10.3390/molecules28062800/s1, Figure S1: Chemical structures of NAMI-A, KP1019 and NKP-1339, Figure S2: Hydrodynamic radius distribution functions of DOTAP and DoHuRu/DOTAP vesicles, Figure S3: UV/Vis absorption spectrum of AziRu in solution, Figure S4: Stern–Volmer plots for the interaction of AziRu with HSA and hTf, Figure S5: Modified Stern–Volmer plots for the interaction of AziRu with HSA and hTf, Figure S6: Fluorescence spectra of HSA and hTf with DOTAP vesicles, Figure S7: Stern–Volmer plots for the interaction of DOTAP and DoHuRu/DOTAP vesicles with HSA and hTf, Figure S8: Hydrodynamic radius distribution functions of DOTAP and DoHuRU/DOTAP vesicles in the presence of HSA, Figure S9: Hydrodynamic radius distribution functions of DOTAP and DoHuRU/DOTAP vesicles in the presence of hTf, Figure S10: CD spectra of hTf in the presence of DOTAP vesicles, Table S1: IC_50_ values of Ru(III) complexes in different tumor and healthy cell lines [82].
