# Supplementary material for: Investigating the Interaction of an Anticancer Nucleolipidic Ru(III) Complex with Human Serum Proteins: A Spectroscopic Study
Source: Molecules. 2023 Mar 20;28(6):2800. doi: 10.3390/molecules28062800 (PMC10055563; doi:10.3390/molecules28062800)
Supplement: Supplementary file 1 [file molecules-28-02800-s001.zip › molecules-2256438-supplementary.pdf]

Supporting information

Table of contents

Figure S1. Chemical structures of NAMI-A, KP1019 and NKP-1339 **page 2**

Figure S2. Hydrodynamic radius distribution functions of DOTAP and DoHuRu/DOTAP vesicles **page 3**

Figure S3. UV/Vis absorption spectrum of AziRu in solution **page 4**

Figure S4. Stern-Volmer plots for the interaction of AziRu with HSA and hTf **page 5**

Figure S5. Modified Stern-Volmer plots for the interaction of AziRu with HSA and hTf **page 6**

Figure S6. Fluorescence spectra of HSA and hTf with DOTAP vesicles **page 7**

Figure S7. Stern-Volmer plots for the interaction of DOTAP and DoHuRu/DOTAP vesicles with HSA and hTf **page 8**

Figure S8. Hydrodynamic radius distribution functions of DOTAP and DoHuRU/DOTAP vesicles in the presence of HSA **page 9**

Figure S9. Hydrodynamic radius distribution functions of DOTAP and DoHuRU/DOTAP vesicles in the presence of hTf **page 10**

Figure S10. CD spectra of hTf in the presence of DOTAP vesicles **page 11**

Table S1. IC_{50} values of Ru(III) complexes in different tumour and healthy cell lines **page 12**

References. **page 13**

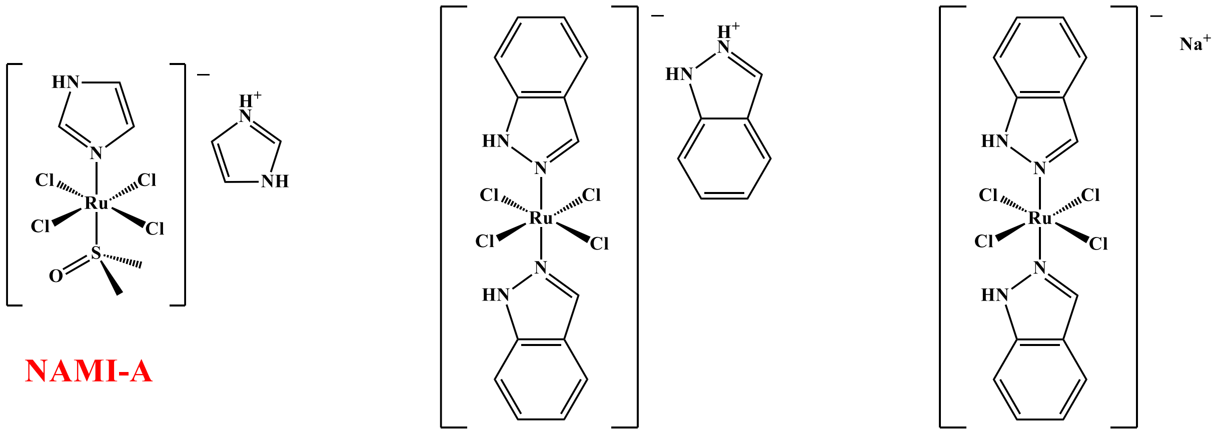

NAMI-A **KP1019** **NKP1339**

Figure S1. Chemical structures of NAMI-A, KP1019 and NKP-1339, as indicated.

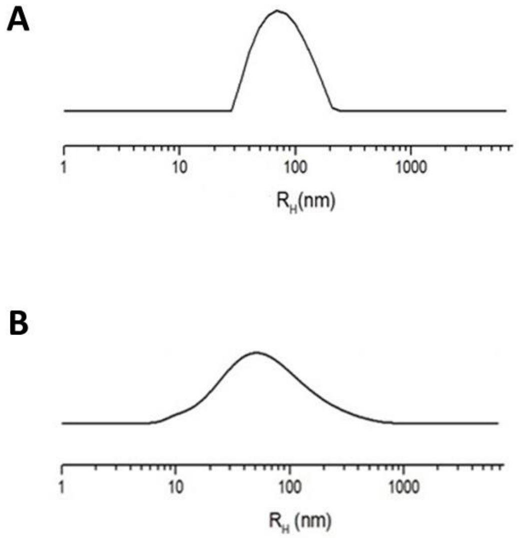

Figure S2. Hydrodynamic distribution functions for (A) DOTAP and (B) DoHuRu/DOTAP vesicles obtained by means of dynamic light scattering (DLS) measurements.

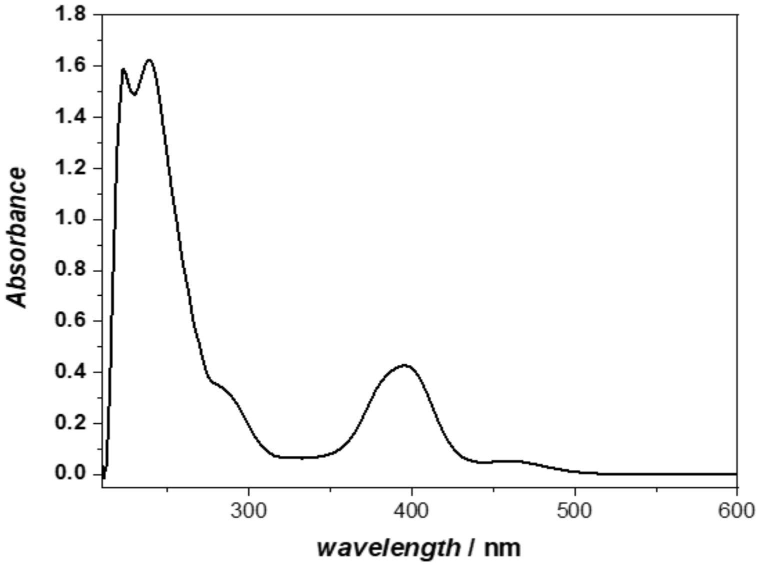

Figure S3. UV/Vis absorption spectrum of AziRu, at the concentration of 300 μM, in PBS buffer, pH 7.4. The spectrum was obtained at the temperature of 20 °C, using a 1-cm path length quartz cuvette.

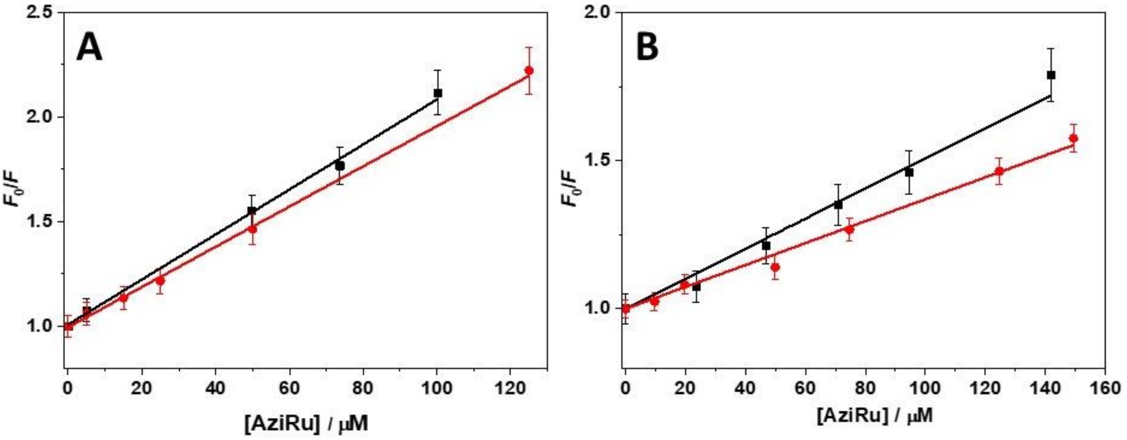

Figure S4. Stern-Volmer plots for the interaction of AziRu with (A) HSA and (B) hTf at 10 °C (black squares) and 20 °C (red circles). The solid lines represent the best fit of experimental data according to equation 1 reported in the main text.

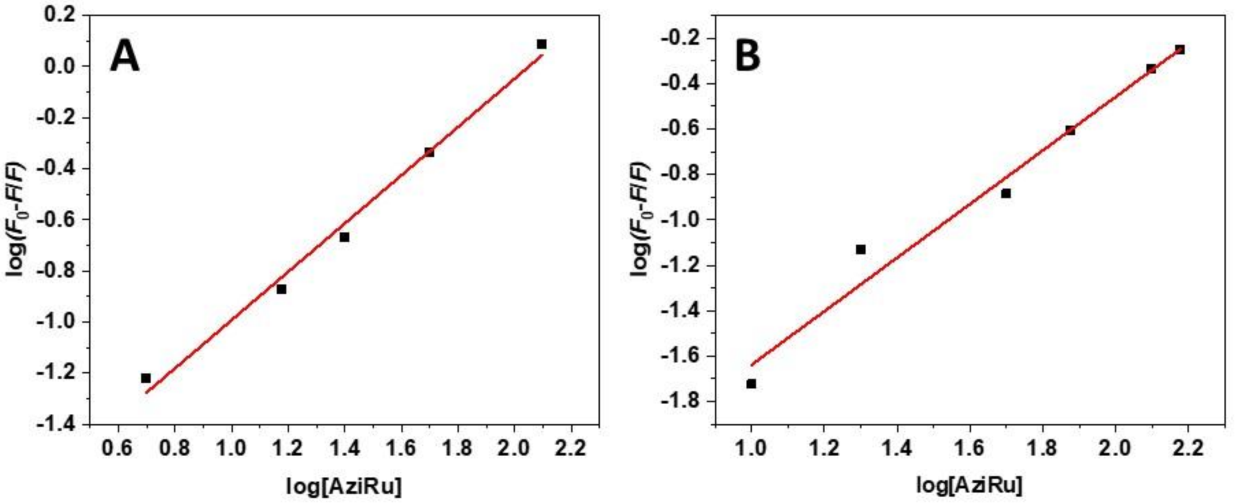

Figure S5. Modified Stern-Volmer plots for the interaction of AziRu with (A) HSA and (B) hTf at 20°C. The solid lines represent the best fit of experimental data according to equation 2 reported in the text. The stoichiometry and the binding constant can be determined from the slope and the intercept of the lines, respectively.

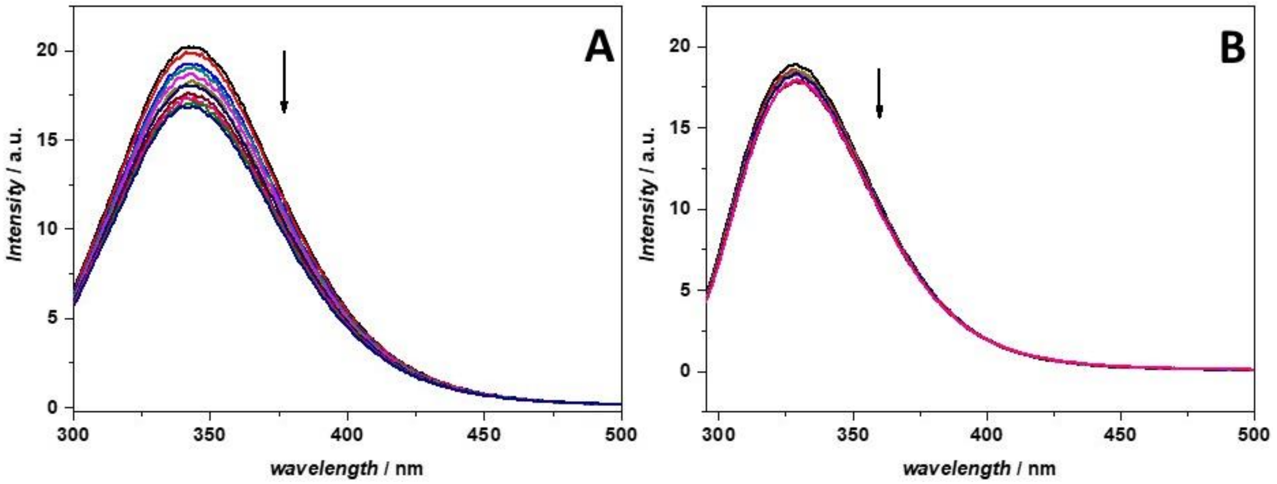

Figure S6. Fluorescence emission spectra of (A) HSA and (B) hTf in the absence (black spectra) and in the presence of DOTAP (colored spectra) vesicles. The arrows indicate the direction of the effects due to increasing concentrations of DOTAP. The excitation wavelength was set at 280 nm. All the experiments were performed in a 1-cm path length quartz cuvette at the temperature of 20 °C.

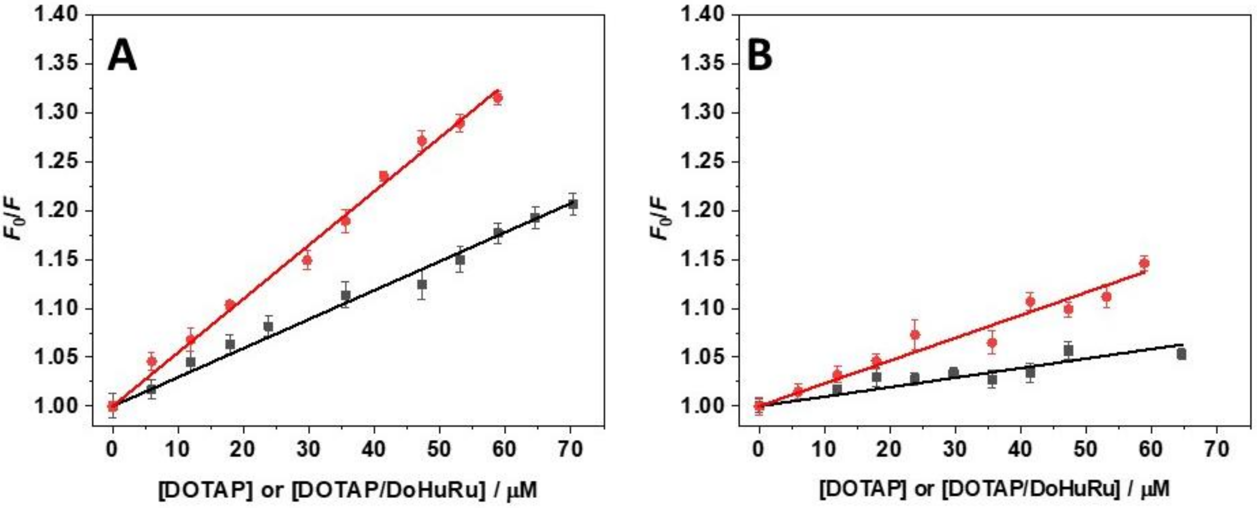

Figure S7. Stern-Volmer plots for the interaction of DOTAP (black squares) and DoHuRu/DOTAP (red circles) vesicles with (A) HSA and (B) hTf at 20 °C. The solid lines represent the best fit of experimental data according to equation 1 reported in the text.

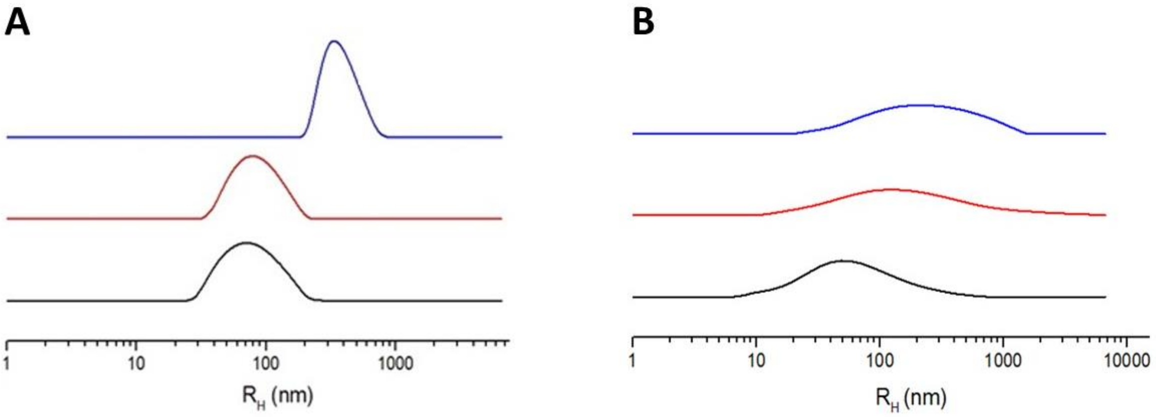

Figure S8. Hydrodynamic radius distribution functions for (A) DOTAP and (B) DoHuRu/DOTAP vesicles obtained by means of dynamic light scattering (DLS) measurements in the absence (black lines) and in the presence of HSA at the concentration of 0.12 µM (red lines) and 0.45 µM (blue lines).

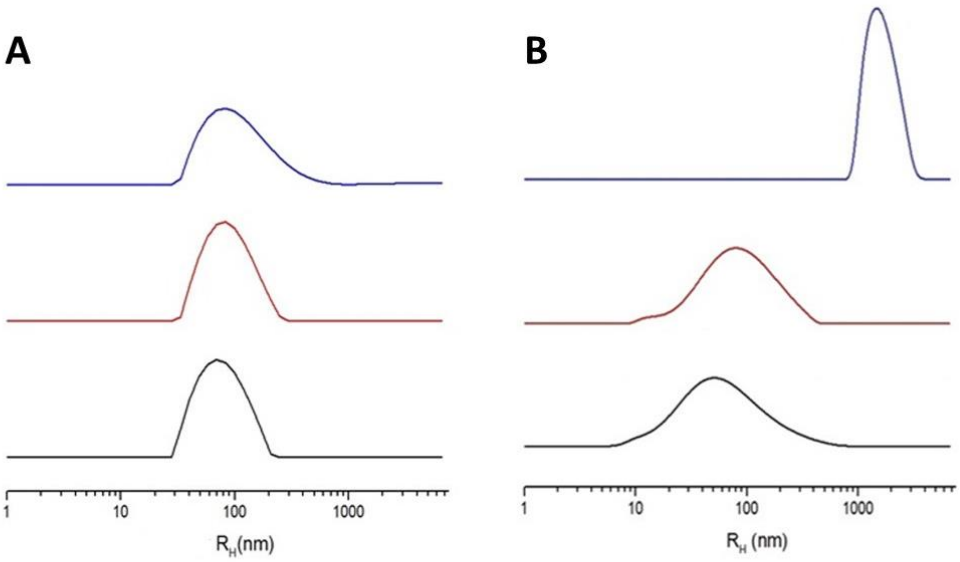

Figure S9. Hydrodynamic radius distribution functions for (A) DOTAP and (B) DoHuRu/DOTAP vesicles obtained by means of dynamic light scattering (DLS) measurements in the absence (black lines) and in the presence of hTf at the concentration of 0.15 μM (red lines) and 0.30 μM (blue lines).

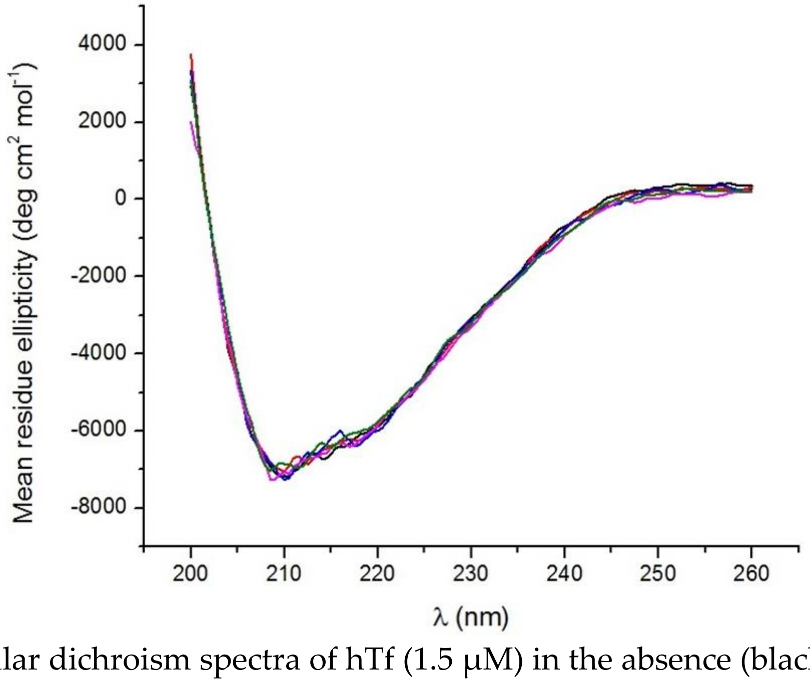

Figure S10. Circular dichroism spectra of hTf (1.5 μM) in the absence (black spectrum) and in the presence of DOTAP vesicles. The concentrations of DOTAP were: 3 μM (red spectrum), 6 μM (blue spectrum), 9 μM (violet spectrum) and 15 μM (green spectrum). The spectra were acquired in 20 mM sodium phosphate buffer, pH 7.4.

Table S1. IC_{50} values (μM) relative to NAMI-A, AziRu and to the effective ruthenium concentration inserted in the DoHuRu/DOTAP liposomes in the indicated cell lines. IC_{50} values are reported as mean ± SEM. MCF-7: epithelial-like breast adenocarcinoma cells; HeLa: human cervical cancer cells; WiDr: human epithelial colorectal adenocarcinoma cells; C6: tumour rat glioma cells; MDA-MB-231, MDA-MB-436, MDA-MB-468 and CG-5: triple-negative breast adenocarcinoma cells.

**IC_{50} (μM)**			
**Cell line**	**NAMI-A**	**AziRu**	**DoHuRu/DOTAP**
**MCF-7**	**620 ± 30**[1]	**305 ± 16**[2]	**10.3 ± 0.2**[3]
**HeLa**	**626 ± 45**[1]	**382 ± 19**[4]	
**WiDr**		**441 ± 20**[2]	**41 ± 10**[5]
**C6**		**318 ± 12**[2]	**34 ± 9**[5]
**MDA-MB-231**		**> 250**[3,6]	**12.1 ± 0.3**[3]
**MDA-MB-436**		**> 250**[3,6]	**20.0 ± 0.2**[3]
**MDA-MB-468**		**> 250**[3,6]	**14.2 ± 0.1**[3]
**CG-5**		**>250**[3,6]	**3.3 ± 0.2**[3]

References:

1. Tan, C.; Wu, S.; Lai, S.; Wang, M.; Chen, Y.; Zhou, L.; Zhu, Y.; Lian, W.; Peng, W.; Ji, L.; et al. Synthesis, structures, cellular uptake and apoptosis-inducing properties of highly cytotoxic ruthenium-norharman complexes. *Dalton Trans.* **2011**, *40*, 8611–8621, doi:10.1039/c1dt10084j.
2. Mangiapia, G.; D'Errico, G.; Simeone, L.; Irace, C.; Radulescu, A.; Di Pascale, A.; Colonna, A.; Montesarchio, D.; Paduano, L. Ruthenium-based complex nanocarriers for cancer therapy. *Biomaterials* **2012**, *33*, 3770–3782, doi:10.1016/j.biomaterials.2012.01.057.
3. Irace, C.; Misso, G.; Capuozzo, A.; Piccolo, M.; Riccardi, C.; Luchini, A.; Caraglia, M.; Paduano, L.; Montesarchio, D.; Santamaria, R. Antiproliferative effects of ruthenium-based nucleolipidic nanoaggregates in human models of breast cancer in vitro: insights into their mode of action. *Sci. Rep.* **2017**, *7*, 45236–45249, doi:10.1038/srep45236.
4. Vitiello, G.; Luchini, A.; D'Errico, G.; Santamaria, R.; Capuozzo, A.; Irace, C.;

Montesarchio, D.; Paduano, L. Cationic liposomes as efficient nanocarriers for the drug delivery of an anticancer cholesterol-based ruthenium complex. *J. Mater. Chem. B* **2015**, *3*, 3011–3023, doi:10.1039/c4tb01807a.

5. Mangiapia, G.; Vitiello, G.; Irace, C.; Santamaria, R.; Colonna, A.; Angelico, R.; Radulescu, A.; D'Errico, G.; Montesarchio, D.; Paduano, L. Anticancer cationic ruthenium nanovectors: from rational molecular design to cellular uptake and bioactivity. *Biomacromolecules* **2013**, *14*, 2549–2560, doi:10.1021/bm400104b.
6. Piccolo, M.; Misso, G.; Ferraro, M.G.; Riccardi, C.; Capuozzo, A.; Zarone, M.R.; Maione, F.; Trifuoggi, M.; Stiuso, P.; D'Errico, G.; et al. Exploring cellular uptake, accumulation and mechanism of action of a cationic Ru-based nanosystem in human preclinical models of breast cancer. *Sci. Rep.* **2019**, *9*, 7006, doi:10.1038/s41598-019-43411-3.
